# The influence of AI literacy on fear of negative evaluation among students and researchers: a multisite cross-sectional study

**DOI:** 10.1186/s12909-026-09721-7

**Published:** 2026-06-16

**Authors:** Fahad Zeed Alanezi, Latifah H. Alenazi, Alwah Mohammed Alkathiri, Adnan Innab

**Affiliations:** 1 Department of Adult Health Nursing, Dr. Sulaiman Al Habib College for Knowledge, Riyadh, Saudi Arabia; 2 Nursing Administration and Education Department, College of Nursing, King Saud University, Riyadh, 14242 Saudi Arabia; 3 Medical Surgical Nursing, College of Nursing, King Saud University, Riyadh, 14242 Saudi Arabia; 4 Nursing Administration and Education Department, College of Nursing, King Saud University, Riyadh, 12372 Saudi Arabia; 5 Department of Health Systems and Nursing Education, Dr. Sulaiman Al Habib College for Knowledge, Riyadh, Saudi Arabia

**Keywords:** Artificial intelligence, Self-efficacy, Educational technology, Students

## Abstract

**Background:**

Generative artificial intelligence (AI) tools are increasingly used in academia to retrieve information, draft manuscripts, and support learning activities. Despite growing research on AI use and literacy in academia, few studies have examined negative perceptions of such use. This study aimed to assess the relationship between AI literacy and fear of negative evaluation among students and researchers in Saudi Arabia.

**Methods:**

A cross-sectional, correlational, descriptive study was conducted among researchers and students from private and public institutions. Data were collected from August to October 2025 using the Meta Artificial Intelligence Literacy Scale and the Brief Fear of Negative Evaluation Scale. Data were analyzed using an independent t-test and multiple linear regression.

**Results:**

Among the 154 participants who responded to the questionnaire, moderate AI literacy and low fear of negative evaluation were reported, with strongest agreement on evaluating AI’s advantages and disadvantages and on solving complex tasks. However, researchers reported significantly stronger concerns than students across multiple items, particularly on items related to being judged when using AI. Age and AI self-efficacy were significant positive predictors of fear of negative evaluation, indicating that older participants and those with higher AI confidence reported slightly higher levels of concern.

**Conclusions:**

This study highlights the need for structured AI education, clear guidelines, and supportive environments to ensure responsible and confident use. Despite the low overall fear of negative evaluation, researchers’ concerns about using AI highlight the importance of supportive environments and clear guidelines. Stronger concern among older and more confident participants indicates the need to reinforce ethical practice and accountability to promote confident, responsible engagement with AI.

**Supplementary Information:**

The online version contains supplementary material available at 10.1186/s12909-026-09721-7.

## Background

Generative artificial intelligence (AI) tools, such as ChatGPT, Google Gemini, and DeepSeek, have rapidly expanded across sectors, including academia, where they are used to retrieve information, assist in drafting manuscripts, and support the development of learning activities [1]. This global shift is also evident in Saudi Arabia, where Saudi Vision 2030 has driven major AI investment and trained over 1 million citizens in AI use [2, 3].

As AI use in academia and research has become increasingly popular, AI literacy has been recommended to help users move beyond simple adoption toward the competencies needed for effective use. AI literacy is considered the foundation of AI competency, enabling students and researchers to effectively use AI tools [4]. Long and Magerko (2020) define AI literacy “as a set of competencies that enables individuals to critically evaluate AI technologies; communicate and collaborate effectively with AI; and use AI as a tool online, at home, and in the workplace” [5]. Furthermore, the components of AI literacy should include the ability to recognize, understand, use, and apply AI tools, as well as to navigate them ethically [6].

However, it is necessary to situate this competency-based view of AI literacy within wider debates about transparency, authenticity, academic integrity, and the disclosure of AI-assisted academic work. Recent studies suggest that students may perceive generative AI as useful for learning support, writing assistance, brainstorming, and research-related tasks, while also expressing concerns about accuracy, privacy, ethical issues, personal development, career prospects, and broader societal values [7]. Recent Saudi educational research further suggests that factors beyond technical ability influence students’ engagement with AI and digital academic tools. AI-supported academic writing has been associated with improved writing performance and sustained student motivation and engagement [8]. Similarly, research on online assessment practices in Saudi Arabia has reported perceived benefits, including increased efficiency, feedback, and convenience, while also identifying technical disruptions, navigation difficulties, and timer-related anxiety among the challenges [9].

AI-use disclosure has become an important academic integrity issue. For example, Gonsalves found that fear of academic repercussions, ambiguous guidelines, inconsistent enforcement, and peer influence affect student non-compliance with AI-use declarations [10]. These concerns are also reflected in emerging discussions of AI shaming, in which AI-assisted academic work may be judged as academically dishonest, less authentic, less valuable, or associated with laziness or deception [11, 12].

Beyond AI literacy, AI use in academic work can also be understood as a social-evaluative behavior, because students and researchers may anticipate judgment about their competence, originality, effort, or academic integrity when AI assistance is disclosed. The fear of negative evaluation is defined as “apprehension about others’ evaluations, distress over their negative evaluations, avoidance of evaluative situations and the expectation that others would evaluate oneself negatively” [13]. Psychological literature identifies fear of negative evaluation as a characteristic feature of social anxiety, reflecting concern about the possibility of being judged negatively by others [14]. In student populations, fear of negative evaluation has been examined as a relevant form of evaluation anxiety in higher education, including among undergraduate nursing students, with a cross-sectional study reporting a moderate level of fear of negative evaluation [15].

Students and researchers may fear negative evaluation when using AI in their research or assignments because institutions have not yet clearly defined the fair and acceptable use of AI in academic work [10, 16]. In this context, disclosure of AI use may itself become an evaluative situation. Students may worry that declaring AI assistance could lead to academic repercussions or negative judgment from others, especially when institutional expectations are unclear or inconsistently enforced [10]. According to the literature, researchers tend to fear disclosing the use of AI in their work [10, 17]. Similarly, they may hesitate to disclose AI assistance if they believe that doing so could lead others to question the validity, authenticity, or value of their work [12]. In addition, concerns about academic integrity and assessment in higher education may complicate the evaluation of AI-assisted work, as markers may find it difficult to distinguish AI-assisted from non-AI-assisted work, and suspected GenAI use may influence marking decisions [18]. Taken together, these issues suggest that fear of negative evaluation in AI-assisted academic work may reflect not only general evaluation anxiety but also impression management concerns about how others perceive one’s academic competence, originality, and ethical standing.

Despite substantial research on AI use in academia, academic integrity, and AI literacy, few studies have examined concerns about judgment when students and researchers use AI. In particular, the relationship between AI literacy and fear of negative evaluation remains underexplored. Furthermore, to the best of our knowledge, no study has explored students’ and researchers’ perspectives regarding the influence of AI literacy on fear of negative evaluation in Saudi Arabia. Therefore, this study aimed to assess the relationship between AI literacy and fear of negative evaluation among students and researchers in Saudi Arabia. The specific aims were to (1) assess the level of AI literacy and fear of negative evaluation, (2) determine the mean differences in fear of negative evaluation according to the participants’ current roles, and (3) determine the influence of AI literacy on fear of negative evaluation.

### Theoretical framework

The self-presentation model of social anxiety guided this study [19]. This model suggests that anxiety occurs when an individual is encouraged to create a preferred impression on an audience, whether real or imaginary, but has doubts about their ability to succeed. Consequently, they anticipate an unpleasant evaluation reaction. The model is appropriate for our study, as the use of AI in academia may increase the fear of negative peer or colleague evaluations (i.e., incompetent, dishonest). Based on the self-presentation model, we propose that AI literacy significantly influences an individual’s fear of negative evaluation.

In the context of AI use in academia, fear of negative evaluation may not arise only from limited competence, but also from concerns about professional identity, academic credibility, ethical responsibility, and the social acceptance of AI-assisted work. Individuals with higher AI literacy and self-efficacy may become more aware of the ethical concerns and possible stigma associated with AI use in academic settings [10, 20]. As a result, improved AI competence may increase concerns about being judged by peers, supervisors, or the broader academic community. This perspective extends the self-presentation model by suggesting that anxiety related to AI use may reflect not only self-doubt, but also greater awareness of professional expectations and social perceptions regarding AI-assisted academic work [19, 21].

## Methods

### Study design and setting

This was a cross-sectional, correlational, descriptive study, which adhered to the Strengthening the Reporting of Observational Studies in Epidemiology (STROBE) checklist.

### Sampling and settings

Convenience sampling was used to obtain a sample of researchers and students in private or government institutions. For this study, students were operationally defined as individuals enrolled in an undergraduate or postgraduate academic program at the time of data collection. Researchers were operationally defined as individuals who self-identified as engaged in academic or scientific research. A participation statement was sent to all eligible participants. To be included in the study, participants had to be (a) 18 years or older, (b) proficient in English, and (c) researchers or students.

The minimum required sample size was 68 based on G*Power 3.1, with a significance level of alpha 0.05, power of 0.8, an effect size of 0.15, and two predictors in a multiple linear regression. The target sample size was increased by 15% to mitigate the impact of missing data, and 154 participants completed the survey.

### Data collection procedure

Data collection was completed over 3 months, from August to October 2025, using a Google Form survey shared via social media platforms such as X and WhatsApp, as well as via email. In addition, active researchers were approached to share the study’s link on their social media accounts. The recruitment statement included all necessary information about the study (i.e., purpose, target population, risks, benefits, and the questionnaire’s anonymity). The survey took approximately 10 min to complete.

### Measurement

The questionnaire comprised three sections. The first section included demographic data on age, gender, access to technology, institution type, field of study, academic rank, and prior attendance at AI training. The main variables were measured using the Meta Artificial Intelligence Literacy Scale (MAILS-short version) and the Brief Fear of Negative Evaluation Scale.

The MAILS-short version was used to assess an individual’s AI skills and perspectives [22]. This tool has 10 items, rated on a 10-point Likert scale, with higher scores indicating greater AI literacy. The scale demonstrated acceptable internal consistency (α = 0.83), and the original authors granted permission to use the tool. As the study used a previously validated instrument to measure medical AI literacy, we did not conduct any further pilot testing [22].

The Brief Fear of Negative Evaluation Scale includes 12 items [23]. Participants had to read each statement and indicate how applicable the characteristic was to them based on a 1–5 scale, with higher scores indicating higher fear of negative evaluation. Participants were recruited from diverse academic contexts, including students and researchers from public and private institutions and different fields of study, to capture variation in AI literacy and fear of negative evaluation across Saudi academic settings. The scale had acceptable internal consistency, ranging from 0.82 to 0.90 [24]. In this study, Cronbach’s alpha was 0.91, indicating that the scale was reliable in this sample. Permission was obtained from the author to use this tool.

### Ethical considerations

Ethical approval was secured from the Institutional Review Board of King Saud University [KSU-HE-25745]. The study strictly adhered to the Declaration of Helsinki. All participants were provided with all necessary information about the study and agreed to take part. Participants were informed that their participation was voluntary and that they had the right to withdraw without consequences. They were also informed that the data would be destroyed after publication of the study. Access to the data was limited to the primary investigator

### Data analysis

The Statistical Package for the Social Sciences (SPSS; version 30) was used for data analysis. For the descriptive statistics, the mean, standard deviation (SD), frequency, and percentage were calculated to describe the demographic data and analyze the items. An independent t-test was used to determine the mean differences in fear of negative evaluation between researchers and students. Levene’s test and histograms were used to assess the assumptions of the t-test (homogeneity of variances and normality). The assumptions of the t-test were met, as the Q-Q plot showed that all observations lay close to the straight line. The final step was to conduct a multiple linear regression to determine the influence of AI literacy on fear of negative evaluation. Linearity was assessed by visual inspection and residuals-versus-fitted-values plots. There was no multicollinearity, as evidenced by the variance inflation factor (VIF) and tolerance values below 5. The independence of observations was checked using the Durbin–Watson statistic (1.761), indicating the absence of autocorrelation.

## Results

### Demographic characteristics of participants

Table 1 presents the demographic and academic characteristics of the participants. A total of 154 participants responded to the questionnaire. The average age was 23.93 years (SD = 7.82), indicating that the majority were graduate students. The sample was mainly female (94.8%), with male participants accounting for only 5.2%. Most participants were affiliated with public institutions (90.9%), and enrolled in health sciences (78.6%), while only around 20% were enrolled in social sciences and humanities. Among academics, lecturers accounted for 22.7% and professors for 18.2%. Slight more than half of the participants had received no prior AI training (51.9%).

**Table 1:** Demographic characteristics of participants (*N* = 154)

Variable (Range)	*n* (%) or M (SD)
Age	23.93 (7.82)
Gender	
Male	8 (5.2%)
Female	146 (94.8%)
Access to Technology	
Limited Access	6 (3.9%)
Mobile Device Only	34 (22.1%)
Personal Laptop	97 (63%)
Institution Provided Device	7 (4.5%)
Shared Device	10 (6.5%)
Institution Type	
Public	140 (90.9%)
Private	12 (7.8%)
Field of Study	
Health Sciences	121 (78.6%)
Social Sciences and Humanities	32 (20.8%)
Attending Training Courses in AI	
No	80 (51.9%)
Yes	71 (46.1%)

### AI literacy and fear of negative evaluation

Table 2 presents descriptive statistics for AI literacy and AI fear of negative evaluation. Overall, participants reported a moderate AI literacy (M = 5.93, SD = 1.87). Based on the scale range, the observed mean score suggests moderate AI literacy among the participants. Item analysis indicated stronger competence in applied AI than in AI development. The highest means items were observed for using AI meaningfully to achieve goals (M = 7.28, SD = 2.96), identifying advantages and disadvantages of AI utilization (M = 7.19, SD = 2.75), the ability to solve complicated tasks when using AI (M = 7.05, SD = 2.77), and dealing with an application based on AI (M = 6.64, SD = 3.03). However, items related to advanced skills were rated less strongly. Specifically, designing new AI applications (M = 3.10, SD = 3.22) and programming new AI applications (M = 3.75, SD = 3.37) were among the weakest.

**Table 2:** Descriptive statistics of AI literacy and fear of negative evaluation

	*N*	M	SD
Overall AI Literacy	154	5.93	1.87
I can tell if I am dealing with an application based on AI.	154	6.64	3.03
I can weigh the consequences of using AI for society.	154	6.11	2.76
I can use AI meaningfully to achieve my goals.	154	7.28	2.96
I can assess what advantages and disadvantages of using AI	154	7.19	2.75
I can program new applications in the field of AI	154	3.75	3.37
I can design new AI applications.	154	3.10	3.22
Although there are often new AI applications, I manage to always be up to date	154	5.74	3.00
I can solve complicated tasks when working with AI.	154	7.05	2.77
I can handle it when interactions with AI frustrate me	154	6.01	2.74
I can prevent an AI from influencing me in my decisions	154	6.45	3.02
Fear of Negative Evaluation	154	2.87	0.597
I am worried about what other people will think of me even when I know it doesn’t make any difference	154	2.84	1.40
I am unconcerned even if I know people are forming an unfavorable impression of me.	154	3.06	1.27
I am frequently afraid of other people noticing my shortcomings.	154	2.88	1.40
I rarely worry about what kind of impression I am making on someone.	154	3.10	1.36
I am afraid others will not approve of me	154	2.69	1.42
I am afraid that people will find fault with me	154	2.80	1.37
Other people’s opinions of me do not bother me.	154	2.88	1.33
When I am talking to someone, I worry about what they may be thinking about me.	154	2.66	1.32
I am usually worried about what kind of impression I make.	154	2.86	1.32
If I know someone is judging me, it has little effect on me.	154	2.91	1.38
Sometimes I think I am too concerned with what other people think of me.	154	2.88	1.44
I often worry that I will say or do the wrong things.	154	2.80	1.40

Regarding the fear of negative evaluation, the overall score was low (M = 2.87, SD = 0.597), indicating that participants were not highly worried about being judged for using AI.

Based on the scale scoring system, the observed mean indicates relatively low fear of negative evaluation among the participants. Item means were closely clustered, ranging from 2.66 to 3.10. Participants were worried about what others might be thinking about them (M = 2.66, SD = 1.32) and about others not approving of them (M = 2.69, SD = 1.42).

### Mean differences of fear of negative evaluation according to the current role of participants

Table 3 presents the mean differences in fear of negative evaluation across participants’ current roles. The results show that researchers consistently reported higher mean scores compared to students. Specifically, researchers reported significantly higher concerns about what others think (M = 3.42, SD = 1.78, *p* = .019), others noticing shortcomings (M = 3.48, *p* = .013), fear of others’ disapproval (M = 3.13, *p* = .029), others finding fault (M = 3.29, *p* = .013), others thinking negatively during conversations (M = 3.23, *p* = .026), and general concerns about the impression made on others (M = 3.35, *p* = .009). In addition, the strongest perceived difference concerned the item related to being judged, where researchers scored higher on the item about being judged for using AI compared to students (M = 3.55, *p* = .002).

**Table 3:** Mean differences of fear of negative evaluation according to the current role of participants

Item Analysis	Groups	*N*	M	SD	Sig.
Total Score		154	2.87	0.597	
I worry about what other people will think of me even when I know it doesn’t make any difference.	Students	123	2.69	1.262	0.019
	Researchers	31	3.42	1.785	
I am unconcerned even if I know people are forming an unfavorable impression of me.	Students	123	2.98	1.109	0.057
	Researchers	31	3.39	1.764	
I am frequently afraid of other people noticing my shortcomings.	Students	123	2.72	1.276	0.013
	Researchers	31	3.48	1.710	
I rarely worry about what kind of impression I am making on someone.	Students	123	3.01	1.277	0.053
	Researchers	31	3.45	1.630	
I am afraid others will not approve of me	Students	123	2.59	1.312	0.029
	Researchers	31	3.13	1.765	
I am afraid that people will find fault with me	Students	123	2.67	1.271	0.013
	Researchers	31	3.29	1.677	
Other people’s opinions of me do not bother me.	Students	123	2.85	1.208	0.292
	Researchers	31	3.03	1.779	
When I am talking to someone, I worry about what they may be thinking about me.	Students	123	2.52	1.104	0.026
	Researchers	31	3.23	1.875	
I am usually worried about what kind of impression I make.	Students	123	2.73	1.139	0.009
	Researchers	31	3.35	1.836	
If I know someone is judging me, it has little effect on me.	Students	123	2.75	1.271	0.002
	Researchers	31	3.55	1.650	
Sometimes I think I am too concerned with what other people think of me.	Students	123	2.76	1.325	0.021
	Researchers	31	3.35	1.780	
I often worry that I will say or do the wrong things.	Students	123	2.69	1.262	0.029
	Researchers	31	3.23	1.839	

### Influence of AI literacy on the fear of negative evaluation

Multiple linear regression was conducted to determine the influence of AI literacy on fear of negative evaluation (Table 4). The overall model was significant [F(5, 142) = 2.73, *p* = .022], explaining only 8% of the variance in fear of negative evaluation (R² =0.08). The relatively small explained variance suggests that additional psychological, institutional, and sociocultural factors that were not examined in the current study may also influence fear of negative evaluation. Age and AI self-efficacy were statistically significant predictors of fear of negative evaluation; however, the observed effects were relatively small and should be interpreted cautiously, given the model’s limited explained variance. Additionally, AI self-efficacy (β = 0.216, *p* = .046) was a significant predictor, indicating that stronger confidence in using AI was associated with slightly higher fear of negative evaluation among this sample. Other subscales (i.e., create AI and AI self-competence) were not significant predictors of fear of negative evaluation (*p* > .05).

**Table 4:** The influence of AI literacy subscales on fear of negative evaluation

Model	Unstandardized Coefficients		Standardized Coefficients	t	*p*-value	95% CI	
						LL	UL
	Β	Std. Error	Β			0.711	3.142
Age	0.023	0.011	0.176	0.124	0.035	0.002	0.043
Create AI subscale	0.011	0.028	0.035	0.411	0.681	− 0.044	0.067
AI Self-Efficacy subscale	0.086	0.043	0.216	0.009	0.046	0.001	0.170
AI Self-Competence subscale	− 0.068	0.040	− 0.168	-1.67	0.095	− 0.147	0.012
AI Literacy subscale	− 0.041	0.049	− 0.094	0.844	0.400	− 0.137	0.055
Model Summary	F(5, 142) = 2.73, R2 = 0.08, *p* = .022						

## Discussion

The study aimed to assess the relationship between AI literacy and fear of negative evaluation among students and researchers in Saudi Arabia. The self-presentation model of social anxiety guided the research, suggesting that anxiety arises when individuals attempt to create a favorable impression on others, but they doubt their ability to create that impression, leading to concerns about negative evaluations by others.

Participants overall reported a moderate level of AI literacy, with their scores indicating their stronger competence in applied AI than in AI development. Previous studies have reported similar trends, indicating that students have competence in using AI due to their knowledge of electronic technology [25, 26]. Furthermore, although 84.5% of students were aware of AI technology, 69.9% lacked formal AI education or training [25]. Despite this gap, 79% favored specialized training programs. A lack of AI-focused curricula and training prevents students from developing technical AI skills, with attention focused on user-facing applications rather than foundations [25].

The overall score for fear of negative evaluation was low, indicating that participants did not feel worried about using AI. This finding reflects the growing acceptance of AI as a supportive tool rather than a replacement for personal effort in both academic and professional settings. However, participants showed some worry about others’ judgments and disapproval. The findings can be explained by uniqueness neglect, the belief that AI cannot account for people’s distinctive situations [27]. Indeed, previous findings suggest that users perceive computerized decision support as less professional [21].

Our findings also indicate that participants acknowledged the numerous benefits of AI, despite both social stigma and integrity concerns. This notion suggests that while the use of AI and its benefits are increasingly acknowledged, the social dynamics and potential stigma associated with its adoption remain significant considerations, particularly in fields that traditionally emphasize human-centered expertise [28]. Such concerns stem from uncertainties about academic integrity and the appropriate use of AI, despite the majority of students reporting greater comfort and convenience when using AI for studying and concept review [29]. The observed hesitation to accept AI, despite its potential advantages, underscores the ethical complexity of AI integration into academic environments, especially regarding originality and intellectual contribution [20, 30]. In addition, previous researchers reported that while AI tools can support information retrieval and academic tasks, concerns remain regarding academic integrity and the reliability of AI-generated outputs [31, 32]. This dilemma suggests that adopting AI in education is also a complex process. In addition, institutional culture and the absence of clear guidelines regarding acceptable AI use may further contribute to concerns about negative evaluation [10, 16]. In academic environments with evolving expectations regarding AI disclosure, originality, and appropriate use, individuals may feel uncertain about how peers, supervisors, or academic institutions will perceive AI-assisted work. Such concerns may increase sensitivity toward criticism, professional judgment, or negative social perceptions associated with AI use in academia [10, 17].

Beyond technical adoption, AI use in academia may also involve professional and social considerations, particularly among researchers, whose credibility is often linked to originality, expertise, and intellectual contribution. Consequently, concerns about how peers and academic communities perceive AI-assisted work may influence individuals’ willingness to openly engage with AI technologies, despite recognizing their practical benefits. This notion may explain why individuals with higher AI competence and awareness still report concerns about negative evaluation.

Researchers reported consistently higher mean scores on fear of negative evaluation than students did across all items, suggesting greater hesitation to use AI. These items included concerns about others’ opinions, the fear that others might notice their shortcomings, and concerns about being judged. This finding suggests that the nature of researchers’ and students’ respective roles may explain the notable differences in their views. Researchers are frequently involved in rigorous scientific work, unlike students, who may view AI tools as learning tools without paying attention to their negative impacts [33]. Therefore, fear of negative evaluation becomes a significant psychological burden for academics, which may, in turn, affect AI use among researchers. Researchers may be more sensitive to AI-assisted work because academic environments often emphasize originality, expertise, critical thinking, and intellectual contribution as central aspects of professional identity. Consequently, the use of AI tools may create concerns regarding how peers and academic communities perceive competence, authenticity, and scholarly credibility. These professional expectations may lead to greater hesitation and vulnerability toward AI use among researchers than among students.

Several differences between students and researchers reached statistical significance, although their magnitudes appeared relatively modest. Therefore, these findings should be interpreted cautiously, as statistical significance does not necessarily indicate strong practical or clinical significance. Nonetheless, the consistent pattern of stronger concern among researchers may still reflect meaningful professional and social sensitivities regarding AI-assisted academic work.

Although the regression model was statistically significant, the explained variance was relatively small, suggesting that additional psychological, institutional, and sociocultural factors beyond AI literacy alone likely influence fear of negative evaluation. Therefore, the predictive relationships identified in this study should be interpreted with caution. Factors such as academic integrity concerns, institutional policies on AI use, trust in AI, disciplinary expectations, and prior experiences with criticism or disclosure may play a significant role in shaping individuals’ concerns about AI-assisted academic work. Previous studies have also suggested that greater knowledge of AI tools may improve individuals’ ability to critically evaluate AI-supported products and recognize the effectiveness of AI integration in educational settings [34]. Future studies are encouraged to examine these broader contextual variables using larger and more diverse samples to provide a more comprehensive understanding of fear of negative evaluation in AI-related academic contexts.

We also found that older participants and those with higher AI self-efficacy reported slightly stronger worry, suggesting a trend toward greater evaluation concern. This pattern should be interpreted cautiously, as it reflects differences in previous exposure to AI, AI literacy, and age. Previous research has shown that age is associated with fear or anxiety about AI tools, with younger users tending to have a more positive attitude toward AI than older users [35, 36]. Our findings indicated that higher self-efficacy was associated with greater fear of negative evaluation, which is inconsistent with previous studies [37, 38]. The findings suggest that greater AI literacy and self-efficacy may increase the awareness of ethical concerns, professional expectations, and potential social scrutiny surrounding AI-assisted academic work. In academic environments with evolving expectations regarding AI disclosure and appropriate use, individuals with higher AI competence may become more sensitive to professional judgment and evaluation concerns [10, 17].

Our positive association may be due to the context of AI self-efficacy, which differs from that reported in the literature. It is worth mentioning that the focus of the previous studies [37, 38] was not on AI. As studies examining the relationship between self-efficacy and fear of negative evaluation in the AI context are lacking, it was not feasible for us to assess its consistency with the literature, warranting further investigation.

### Limitations and recommendations for future research

Despite our study’s significant novelty, it faced several limitations. Our study used a cross-sectional design and a self-report approach to measure fear of negative evaluation, which limits consideration of underlying factors that may influence students and researchers. This limitation is important, given that fear of negative evaluation is considered a psychological phenomenon. Second, the use of a convenience sampling method enhanced recruitment but could limit the generalizability of the findings, as the sample may not be representative of the population. The use of such a sampling method may have increased the risk of self-selection bias, as individuals with stronger interest, familiarity, or positive attitudes toward AI may have been more likely to participate. Therefore, the findings may not fully represent students and researchers with limited exposure to or interest in AI technologies. Third, the imbalance between students and researchers may increase the risk of a type II error. Thus, future researchers are recommended to collect data from a comparable sample using such a robust design and sampling method to enhance the generalizability of the findings.

Another limitation is the gender imbalance within the sample, with female participants comprising the majority of respondents. This imbalance may have influenced findings on AI literacy and fear of negative evaluation, as previous research suggests gender differences in technology confidence, AI perceptions, and anxiety-related responses [35, 36]. Moreover, the regression model explained only a limited proportion of the variance in fear of negative evaluation, indicating that the study did not capture other relevant psychological, institutional, and sociocultural factors. Therefore, the findings should be interpreted with caution.

### Implications for education and practice

Our findings present several practical implications for education and practice.

First, the moderate level of AI literacy indicates the need to strengthen structured AI education within the curriculum, not only focusing on how to use these tools but also on understanding their limitations and ethical considerations. This approach can help students move beyond basic use toward more informed and responsible engagement with AI. Although the majority of participants demonstrated stronger self-efficacy in using AI for practical tasks, targeted intervention and training are required to strengthen the conceptual understanding of AI usability.

Second, although fear of negative evaluation was generally low, researchers expressed some concerns about AI use, indicating the need for a more supportive environment in which AI use is openly discussed and clearly guided. Clear policies and transparent guidelines can help reduce uncertainty and make individuals feel more comfortable using AI properly.

Finally, the finding that older participants and those with higher confidence in AI reported stronger concern suggests that awareness of professional expectations may heighten sensitivity to judgment. This notion highlights the importance of reinforcing ethical use, accountability, and proper disclosure, enabling researchers to use AI confidently without worrying about negative perceptions.

## Conclusion

This study provides evidence of AI literacy and fear of negative evaluation among students and researchers, suggesting a growing acceptance of AI use in academic contexts.

However, concerns about being judged and the significant influence of age and AI self-efficacy highlight the persistent apprehension toward AI use. These findings demonstrate the importance of integrating structured AI education, clear guidelines, and supportive environments within education and practice. Strengthening both competence and confidence in the ethical and appropriate use of AI is essential to ensure its effective and responsible adoption in the academic context.

## Supplementary Information

Supplementary Material 1.

## Data Availability

The data supporting the findings of this study are available from the corresponding author [FA] upon reasonable request.
